# Co-occurrence of genomic imbalances on Xp22.1 in the *SHOX* region and 15q25.2 in a girl with short stature, precocious puberty, urogenital malformations and bone anomalies

**DOI:** 10.1186/s12920-018-0445-8

**Published:** 2019-01-09

**Authors:** Alice Monzani, Deepak Babu, Simona Mellone, Giulia Genoni, Antonella Fanelli, Flavia Prodam, Simonetta Bellone, Mara Giordano

**Affiliations:** 1 Division of Pediatrics, Department of Health Sciences, Università del Piemonte Orientale, Via Solaroli 17, 28100 Novara, Italy; 2 Laboratory of Human Genetics, Department of Health Sciences, Università del Piemonte Orientale, Via Solaroli 17, 28100 Novara, Italy

**Keywords:** Short stature, *SHOX*, Enhancers, CGH array

## Abstract

**Background:**

Mutations of *SHOX* represent the most frequent monogenic cause of short stature and related syndromes. The genetic alterations include point mutations and deletions/duplications spanning both *SHOX* and its regulatory regions, although microrearrangements are confined to either the downstream or upstream enhancers in many patients. Mutations in the heterozygous state have been identified in up to 60–80% of Leri-Weill Dyschondrosteosis (LWD; MIM #127300) and approximately 4–5% of Idiopathic Short Stature (ISS; MIM#300582) patients. Homozygous or compound heterozygous mutations as well as biallelic deletions of *SHOX* and/or the enhancer regions result in a more severe phenotype, which is known as Langer Mesomelic Dysplasia (LMD; MIM #249700).

**Case presentation:**

A 17 year old girl, presented with severe short stature, growth hormone deficiency (GHD), precocious puberty, dorsal scoliosis, dysmorphisms and urogenital malformations. She was born with agenesis of the right tibia and fibula, as well as with a supernumerary digit on the left foot. Array comparative genomic hybridization (aCGH) analysis detected the presence of two distinct duplications on Xp22.1 flanking the *SHOX* coding sequence and involving its regulatory regions. An additional duplication of 1.6–2.5 Mb on 15q25.2 that included 13 genes was also identified. The girl was adopted and the parent’s DNA was not available to establish the origin of the chromosome imbalances.

**Conclusions:**

The complex phenotype observed in our patient is probably the result of the co-occurrence of rearrangements on chromosomes Xp22.1 and 15q25.2. The duplicated region on 15q25.2 region is likely to contain dosage-sensitive genes responsible for some of the clinical features observed in this patient, whereas the extreme short stature and the skeletal anomalies are likely attributable to the comorbidity of GHD and copy number variants in the *SHOX* region.

**Electronic supplementary material:**

The online version of this article (10.1186/s12920-018-0445-8) contains supplementary material, which is available to authorized users.

## Background

The presence of multiple independent genomic imbalances in the same patient might result in complex phenotypes due to either an additive or interactive effect of single rearrangements. Here, we report the case of a patient carrying two apparently distinct duplications on Xp22.1 that, involve the *SHOX* (Short Stature homeobox-containing gene, MIM *312865) region and a duplication on 15q25.2 that likely contributes to the the determination of her clinical features.

Molecular defects of *SHOX*, which is located on pseudoautosomal region 1 (PAR1), include intragenic mutations, complete or partial deletions/duplications of the coding sequence and/or the enhancer regions [1] that result in conditions that are generally characterized by mild to severe short stature. Haploinsufficiency of the *SHOX* gene can result in Lerì-Weill Dyschondrosteosis (LWD; MIM #127300) with disproportionate short stature, mesomelia and Madelung deformity of the wrist as well as tibial bowing. Loss of both copies of *SHOX* cause Langer mesomelic Dysplasia (LMD; MIM #249700), which is a condition with extreme short stature, severe shortening or aplasia of the ulna and fibula, as well as boththickening and curvature of the radius and tibia. *SHOX* mutations are also present in approximately 2–10% of Idiopathic Short Stature (ISS) patients, depending on which study cohort is considered [2].

Genomic rearrangements involving chromosome 15q25.2 constitute a rare finding. Nevertheless, there have been some reports of copy number variations (CNVs) within this region associated with recognizable phenotypes. In most cases, these mutations were represented by deletions, whereas 15q25.2 microduplications have been described in only three previous cases [3, 4] and a few patients who were reported on in the DECIPHER database (https://decipher.sanger.ac.uk/).

## Case presentation

The patient was referred to our third-level Paediatric Endocrinology Unit when she was 9 years old for short stature and suspected precocious puberty. She was of Russian origin and was adopted at the age of 2 years. Limited data were available about her family and perinatal history. The biological mother was affected by insulin-dependent diabetes mellitus and arterial hypertension. Nothing was known about the biological father. No data were available about gestational age, neonatal weight and length as well as general conditions at birth. She was born with agenesis of the right tibia and fibula, and underwent mid-thigh amputation when she was 5 years old. She also presented a supernumerary digit of the left foot, which was excised when she was 8 years old.

**Fig. 1:**
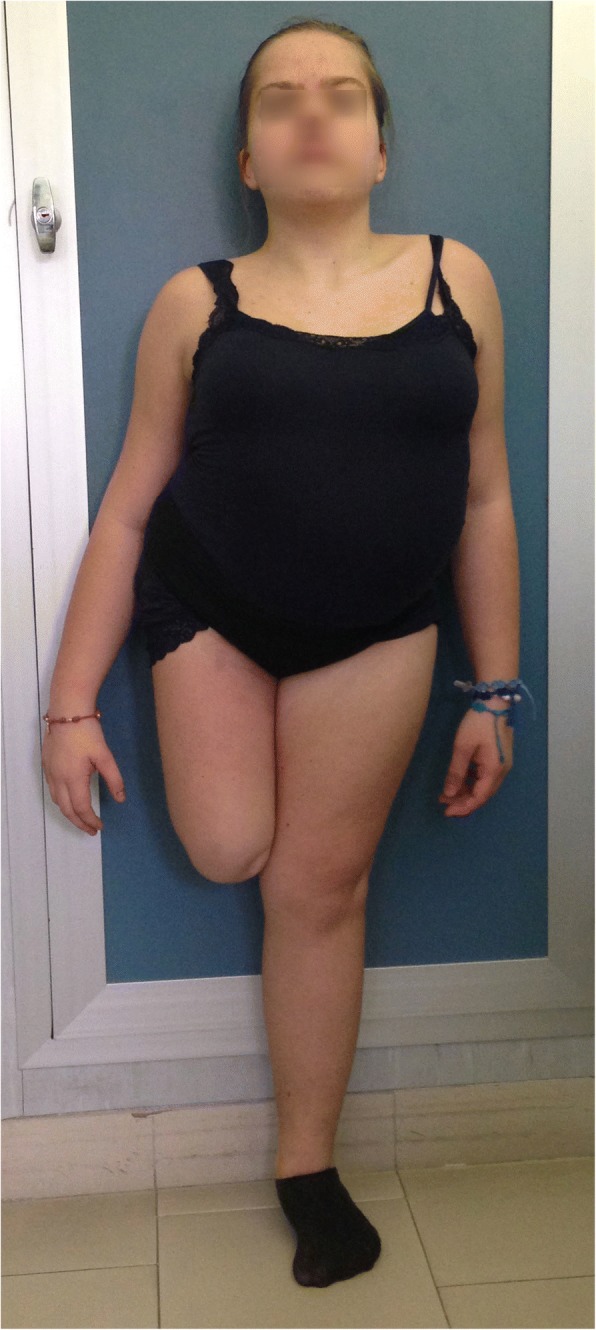
Clinical phenotype of the girl: dorsal scoliosis, short neck, mid-thigh amputation of right leg for agenesis of tibia and fibula

At the age of 9 years, she was referred to our Paediatric Endocrinology Unit for short stature and with potential precocious puberty. Her height was <3rd percentile on CDC growth charts for sex and age (parental target was not known), her weight was in the 25th percentile (http://www.cdc.gov/growthcharts) with a body mass index in the overweight range according to International Obesity Task Force cut-offs [5]. The pubertal stage was a B3 PH2 according to Tanner criteria. She had dorsal scoliosis, a short neck, upturned nose and hypotelorism (Fig. 1). Her systolic and diastolic blood pressure values were in the 90th percentile for sex and height. Biochemical evaluations excluded celiac disease and revealed a normal blood cell count, normal thyroid, kidney, liver and adrenal function. The levels of LH/FSH were > 1 (LH 8.6 mIU/ml, FSH 7.5 mIU/ml) with pubertal oestrogens level, normal fasting insulin and glucose levels with normal glycosylated haemoglobin. Growth hormone (GH) secretion, which was evaluated by Arginine and L-Dopa provocative tests (2.5 ng/ml and 6.8 ng/ml, respectively), revealed a GH deficiency. Cerebral magnetic resonance (MR) showed that her pituitary gland had anormal size, morphology and contrastographic characteristics. Echocardiography and abdominal ultrasonography were performed, and there were no pathological findings. In particular, pelvic ultrasonography showed a uterus with an apparent normal size and morphology as well as a thin endometrium and ovaries with an initial follicular pattern, but the reliability of the exam was limited by bowel gases.

Treatments with human recombinant GH and triptorelin were started to achieve the best growth gain. Triptorelin was able to block the puberty progression. At 14 years, triptorelin was withdrawn, and, at 15 years, menarche appeared. Her final height was 137.5 cm, which corresponds to <3rd percentile.

When she was 17 years old, she complained severe abdominal pain during a menses. A pelvic ultrasonography was performed revealing a picture of hematocolpos. The abdomen MR showed uterus didelphys with double vagina. Due to the presence of these uro-genital anomalies, arterial hypertension at a young age and the family history for insulin-dependent diabetes, molecular analyses of the HNF-1β gene was performed. No alteration was found in this gene.

**Fig. 2:**
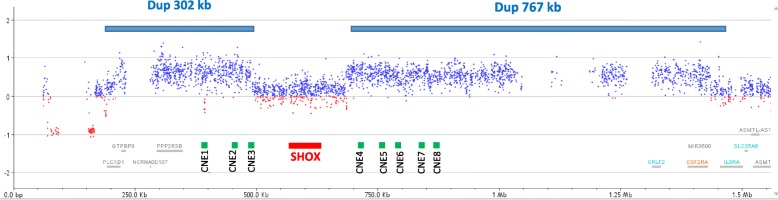
Array-CGH profile performed with a high resolution array containing 8072 probes within the PAR1 region. The two gains (blue bars) flanking the *SHOX* coding region from Xp22.33 192,136–494,191 × 3 and 686,753–1453,835 × 3 (NCBI build 37, hg 19) respectively, are shown. The unbalances include the CNE elements corresponding to the upstream (CNE1–3) and downstream (CNE1–8) enhancers

**Fig. 3:**
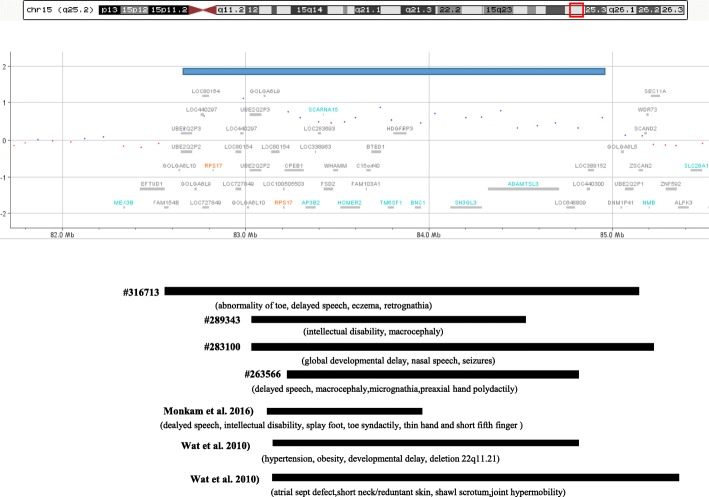
Graphic representation of the duplication of chromosome 15q25.2 (blue bar) with known genes included in this region. Other duplications reported in the Decipher database and in literature are shown. The Decipher ID or the reference is reported for each patient. Clinical features are shown in parenthesis below the corresponding duplication

Because of the complexity of the case with multi-system involvement, array comparative genomic hybridization (aCGH) analysis was performed using the standard Agilent Human Genome G3 SurePrint 8x60K Microarray (Agilent Technologies, California, USA). Two distinct duplications flanking the *SHOX* gene on Xp22.1 (Fig. 2) and an additional duplication of 1.6–2.5 Mb on 15q25.2 (Fig. 3) were detected. To better characterize the Xp22.1 imbalances, confirmed by MLPA, a custom CGH array was used with a high probe density (371 bp average probe spacing) in the PAR1 region (Fig. 2). This analysis revealed the presence of a triple dose of two distinct segments of 302 Kb (chrX:192,136–494,191 × 3, NCBI build 37, hg 19) and 767 Kb (chrX:686,753–1453,835 × 3) that included the upstream and downstream enhancer regions respectively, whereas the *SHOX* coding gene was present in two normal copies (Fig. 2).

The presence of the 15q25.2 duplication was confirmed through MLPA (Multiplex Ligation Probe Amplification) with a probe specifically designed within the *CPEB1* gene. The 15q25.2 duplicated region was examined using the Human resource websites (https://www.ncbi.nlm.nih.gov/projects/genome/guide/human/) at NCBI and the Archive EnsEMBL (https://www.ensembl.org/info/website/archives/index.html). This region contains 13 genes, some of them with a known function (Additional file 1: Table S1). Three of these, namely *RPS17, CPEB1* and *HOMER,* have been previously associated to well defined disorders albeit none of these is apparently related to the clinical features observed in our patient.

Unfortunately, the biological parents were not available to establish the origin of the duplications.

We performed a search in DECIPHER database (https://decipher.sanger.ac.uk/) and identified 4 patients carrying similar micro-duplications (Fig. 3).

## Discussion and conclusion

We found imbalances in the *SHOX* region and on 15q25.2 in a girl with short stature, precocious puberty, urogenital malformation and bone anomalies of the limbs.

The duplicated 15q25.2 region of 1.6–2.5 Mb includes 13 OMIM genes (Fig. 3 and Additional file 1: Table S1) among those, *RPS17* and *HOMER2* cause two Mendelian disorders, including Diamond-Blackfan Anemia 4 (DBA4) and autosomal dominant hearing loss, respectively. DBA4 is a recessive condition caused by biallelic loss of function mutations, whereas *HOMER2* was found to be mutated only once in a family in which deafness co-segregated with a missense mutation [6]. However, the effect of a duplications of these two genes remains unknown as well as its implication in the patient’s phenotype. Patients with similar duplications at 15q25.2 have rarely been reported in the literature [3, 4] and few cases are present in the DECIPHER database that present clinical features resembling those observed in our patient such as anomalies of the foot digits, hypertension, and a short neck (Fig. 1).

One of the genes included in the 15q25.2 duplicated region is *CPEB1*, and the deletion of *CPEB1* is associated with premature ovarian insufficiency, which is characterized by primary amenorrhea [7]. Conversely, we can speculate that *CPEB1* duplication may account for the precocious puberty observed in our patient.

The extreme short stature might be the result of the co-occurrence of two independent causes: GHD and rearrangements in the *SHOX* region. Several patients with *SHOX*–deficiency related phenotypes have been described carrying duplications of either upstream or downstream conserved noncoding elements (CNEs) that function as enhancers of *SHOX* transcription. In the present patient, the aCGH identified two gains flanking *SHOX,* including all the upstream and downstream CNEs (Fig. 2) as well as a normal double dose of the *SHOX* coding sequence. However, aCGH did not allow us to establish if the two gains correspond to duplications lying *in cis* on the same chromosome affecting the expression of only one *SHOX* copy, or if there are two duplications carried *in trans* by the two different X-chromosomes. This latter situation should affect the expression of both copies of *SHOX*. Another possible explanation might be the presence of one uninterrupted duplication on one X-chromosome extending from 192,136 to 1453,835 on a length of 1262 Mb and a deletion of approximately193 Kb, including the *SHOX* coding sequence from 494,191 to 686,753 on the other X that compensates for the increased dose of *SHOX*. Additionally in this case, the patient would be compound heterozygous for two *SHOX* alterations. Homozygous or compound heterozygous defects of *SHOX* or its enhancer region cause Langer mesomelic “dysplasia” (LMD) (OMIM 249700), which is a rare recessive condition characterized by severe short stature, mesomelic and rhizomelic dysgenesis of the limbs involving hypoplasia or aplasia of the ulna and fibula. Our patients did not exhibit a typical LMD phenotype; however, in addition to the severe short stature, she was born with agenesis of the right tibia and fibula. Hypoplastic or absent proximal half of the fibula has been described among the principal clinical features of LMD [8], and it is thus conceivable that, in our patient, the expression of both *SHOX* alleles was altered.

In conclusion, the complex phenotype of our patient, including severe short stature and skeletal anomalies, was probably the result of rearrangements in the *SHOX* regulatory region exacerbated by GH deficiency. The duplication on 15q25.2 might have contributed to some of the clinical features through the action of unidentified dose-sensitive gene/s.

## Additional file

Additional file 1: **Table S1.** Genes with a known function included in the 15q25.2 duplication. (DOCX 18 kb)

## Abbreviations

aCGH: array Comparative Genomic Hybridization
CNE: Conserved Noncoding Elements
CNVs: Copy Number Variations
DBA4: Diamond-Blackfan anemia 4
GH: Growth Hormone
LMD: Langer Mesomelic “Dysplasia”
MLPA: Multiplex Ligation Probe Amplification
MR: Magnetic Resonance
PAR1: Pseudoautosomal Region 1
