# Supplementary material for: Co-occurrence of genomic imbalances on Xp22.1 in the SHOX region and 15q25.2 in a girl with short stature, precocious puberty, urogenital malformations and bone anomalies
Source: BMC Med Genomics. 2019 Jan 9;12:5. doi: 10.1186/s12920-018-0445-8 (PMC6327496; doi:10.1186/s12920-018-0445-8)
Supplement: Supplementary file 1 — Table S1. Genes with a known function included in the 15q25.2 duplication. (DOCX 18 kb) [file 12920_2018_445_MOESM1_ESM.docx]

| **Gene** | **Product** | **Function** | **Disease in which the gene is involved** |
| --- | --- | --- | --- |
| ***RPS17*** | Ribosomal Protein S17 | Component of the ribosome 40S subunit | Diamond-Blackfan anemia 4  (autosomal recessive) |
| ***CPEB1*** | Cytoplasmic Polyadenylation Element Binding Protein 1 | RNA binding protein that regulates the translation during oocyte maturation | the deletion is associated to POI (Premature Ovarian Insufficiency) |
| ***AP3B2*** | Adaptor Related Protein Complex 3 Beta 2 Subunit | Involved in the neurotransmitter release from neurosecretory vesicle | none |
| ***SCARNA15*** | Non coding RNA,  Small Cajal Body-Specific RNA 15 | May play a role in the formation of spliceosomal small nuclear RNA | none |
| ***FSD2*** | Fibronectin Type III And SPRY Domain Containing 2 | unknown | none |
| ***WHAMM*** | (WHDC1) was protein homolog associated with actin, golgi membranes, and microtubules | Mediates Golgi membrane association an actin polymerization | none |
| ***HOMER2*** | Homer Scaffolding Protein 2 | Postsynaptic scaffolding protein involved in maintaining neuronal plasticity at glutamatergic synapses | Autosomal dominant hearing loss |
| ***BTBD1*** | BTB Domain Containing 1 | Probable substrate-specific adapter of an E3 ubiquitin-protein ligase complex which mediates the ubiquitination and subsequent proteasomal degradation of target proteins | none |
| ***TM6SF1*** | membrane protein transmembrane 6 superfamily 1 | Lysosomal protein, may function as sterol isomerase | none |
| ***HDGFRP3*** | Hepatoma-Derived Growth Factor, Related Protein 3 | Enhances DNA synthesis and may play a role in cell proliferation | none |
| ***BNC1*** | Basonuclin 1 | Zinc finger transcriptional factor essential for mouse spermatogenesis | none |
| ***SH3GL3*** | SH3-domain GRB2-like 3 | In zebrafish it regulates endocytosis in the developing vasculature | none |
| ***ADAMTSL3*** | A Disintegrin-Like And Metalloprotease Domain With Thrombospondin Type I Motifs-Like 3 | It might be involved in cell-cell or cell-extracellular matrix adhesion or regulation of ADAMTS protease functions | rs950169 associated with Schizophrenia |
